# Exploring patient-reported outcomes to assess progress in inpatient low vision rehabilitation

**DOI:** 10.1186/s41687-025-00960-8

**Published:** 2025-11-18

**Authors:** E. P. J. van Munster, A. B. M. Schilderman, R. M. A. van Nispen, A. A. J. Roelofs, A. M. Bootsma, H. P. A. van der Aa

**Affiliations:** 1 Amsterdam UMC Location Vrije Universiteit Amsterdam, Ophthalmology, De Boelelaan 1117, Amsterdam, The Netherlands; 2 Amsterdam Public Health, Quality of Care, Mental Health, Aging and Later Life, Amsterdam, The Netherlands; 3 Robert Coppes Foundation, Expertise Innovation and Knowledge, Vlasmeersestraat 38-A, Vught, The Netherlands; 4 Royal Dutch Visio, Het Loo Erf, Centre of Expertise and Inpatient Rehabilitation for Blind and Partially Sighted People, Waldeck-Pyrmontstraat 31, Apeldoorn, The Netherlands; 5 The Lighthouse Guild NYC, 250 W 64th St, New York, USA

**Keywords:** Low vision, Vision impairment, Inpatient low vision rehabilitation, Patient-reported outcomes, (cost-) effectiveness

## Abstract

**Background:**

To date, (cost-)effectiveness of multidisciplinary inpatient low vision rehabilitation (ILVR) programs is unknown and adequate patient-reported outcome measures (PROMs) are lacking. The purpose of this exploratory study was to identify relevant patient-reported outcomes (PROs) within ILVR, personal, contextual and procedural factors affecting ILVR outcomes, and PROMs implementation preferences.

**Methodology:**

Semi-structured interviews were conducted among adults with vision impairment (VI) who follow(ed) an ILVR program between January 2020 and January 2022 (*n* = 19). Healthcare providers of each professional group were interviewed as well (*n* = 10). Tailored interview guides were used to explore PROMs in ILVR from an early development stage. Interview data was sorted into categories of relevant PROs, personal, contextual and procedural factors, and implementation suggestions.

**Results:**

Participants indicated seven potential PROs: mindset, knowledge and strategies, practical skills, self-reliance, social participation, relationships and personal development. Personal, contextual and procedural factors affecting these PROs were related to patient characteristics, coping mechanisms, social support, rehabilitation circumstances, quality of care and transfer to the home environment. Implementation suggestions such as modes of administration, frequency, burden and customization were discussed.

**Conclusions:**

This exploratory study provides the first step toward future development and implementation of PROMs in ILVR. Further establishment of a set of PROMS requires an extensive process of matching the themes with accurate PROMs, in which it seems generic and specific PROMs for individuals with VI can be effectively combined. Moreover, the accessibility and the burden on patients and healthcare providers should be taken into account, for example by using different modes of administration and computer adaptive testing.

**Supplementary Information:**

The online version contains supplementary material available at 10.1186/s41687-025-00960-8.

## Background

Worldwide, around 338 million individuals are blind or have a moderate or severe vision impairment, and these estimates are expected to increase by almost 200 million by the year 2050 [1]. Vision impairment (VI) reduces the ability to perform daily activities, it affects participation, e.g. reading, mobility, computer work and leisure activities, and is associated with deterioration in physical health, mental health, quality of life and life satisfaction [2–4]. Low vision rehabilitation can be offered to strengthen (functional) skills associated with participation, and improve emotional wellbeing and quality of life [5–10]. 

In the Netherlands, where approximately 300,000 individuals have VI [11, 12], low vision care is widely available in outpatient settings, but only one organization offers inpatient low vision rehabilitation (ILVR). This is an intensive program of seven months on average, during which individuals with VI stay at the facility for three to five days per week to work on rehabilitation goals. Some of them also have severe auditory impairment, are non-native speakers, or have acquired brain injury. Patients are able to improve several practical skills, but are often also in need of psychological support related to adaptation and reevaluation of their situation and cognitions. Therefore, the program includes a variety of training programs, such as low-vision training, occupational therapy, braille, mobility training, computer training, creative and music therapy, social work and psychological therapy. Before starting ILVR, an eligibility assessment is conducted to assess whether ILVR seems appropriate for the patient. If deemed appropriate, a week of observation is conducted to explore the patient’s rehabilitation needs, motivation, circumstances, capabilities and potential. This information is used to define the rehabilitation goals in consultation with trainers and compiled into a rehabilitation plan by a psychologist. During the ILVR program, this rehabilitation plan is evaluated every six weeks during a multidisciplinary meeting.

Earlier research has shown that patients in the Dutch ILVR program showed improvements in mental health and independence after one year [13]. However, the effectiveness of ILVR remains insufficiently supported by scientific evidence. While multidisciplinary rehabilitation for patients with VI has been shown to enhance participation and quality of life [8, 9, 14], the effects on vision-related and health-related quality of life, as assessed in randomized controlled trials, have generally been modest [7]. Additionally, a retrospective comparative study conducted in the United States indicated that ILVR of 4 to 6 weeks was more effective in improving visual (motor) functioning and mobility compared to a low-vision outpatient rehabilitation program, but with an additional expenditure of 39,000 USD per patient [15]. 

Patients and their social environment, as well as healthcare providers and health insurance companies, could benefit from (cost-)effective ILVR trajectories. This aligns with the World Health Organization’s (WHO) recommendation that individuals with irreversible VI should be supported by the government to overcome their barriers to participate in life, while care and rehabilitation programs and services are continuously evaluated and strengthened [16]. Successful rehabilitation programs ensure that patients are able to translate and embed learned skills into their own environment (effectiveness) in as little time and energy as possible (efficiency), leading to cost-effective programs that improve participation and quality of life. These programs might also reduce healthcare costs and loss of income, costs that are higher in individuals with VI than in those without [17]. The expected increase in the number of individuals with VI emphasizes the importance of evaluating and improving (cost)-effectiveness [1]. 

Lately patient-reported outcome measures (PROMs) are more frequently used to evaluate effectiveness of interventions in clinical practice, and also to monitor the patients’ desired treatment outcomes [18, 19]. PROMs help to measure the patient’s perspective on (health)conditions, such as functioning, participation restrictions, and quality of life [20]. These measures can be classified as generic PROMs intended for general use, and condition-specific PROMs for patients who have a specific medical condition [21]. Multiple reviews have been conducted to provide an overview of the PROMs used in individuals with VI, sometimes tailored to specific eye-diseases, e.g. glaucoma, macular degeneration, retinal diseases and VI after a stroke [22–31]. 

To evaluate and improve (cost-)effectiveness of ILVR programs by using PROMs, there is a need to measure the most relevant constructs, or ‘patient-reported outcomes’ (PROs), during ILVR. An abundance of outcome measures are used in evaluating effectiveness of low vision rehabilitation (LVR) [10, 24], however, relevant PROs and PROMs for ILVR have not yet been established. Furthermore, there is a call to analyze, strengthen and optimize rehabilitation programs [16]. Previous studies showed patient characteristics, as well as and physical, psychological and emotional factors can affect the use of low vision aids, visual ability, and quality of life [13, 32, 33]. However, specific factors influencing outcomes in ILVR remain unknown. To meaningfully evaluate the (cost)-effectiveness with PROMs in ILVR, it is essential to monitor factors that limit or enhance effectiveness, as these may explain variations in progress during individual rehabilitation trajectories. Awareness of relevant PROs, together with these associated factors, provides an opportunity to develop a set of PROMs tailored for ILVR. Such PROMs can support the determination and improvement of the (cost-)effectiveness of care provided within ILVR.

Nevertheless, introducing PROMs in healthcare organizations is not straightforward. Barriers to incorporate PROMs in practice are frequently reported at both individual and organizational levels [34–36]. Implementation of PROMs can be enhanced by matching the needs and preferences of patients and healthcare providers, creating a positive attitude, and anticipating on organizational changes associated with adopting PROMs [34, 37, 38]. 

Given these challenges, the present study takes an exploratory approach to identify relevant PROs within ILVR, to investigate personal, contextual and procedural factors influencing ILVR outcomes, as well as to explore PROM implementation from the perspectives of both patients and healthcare providers. This exploratory study serves as a foundational step toward the future development and validation of a set of PROMs tailored specifically for ILVR.

## Methods

The Medical Ethical Committee of Amsterdam UMC, location VUmc, the Netherlands, assessed the study protocol and provided exemption of ethical approval (ref: 2021.0326). This study was performed according to the standards of the Declaration of Helsinki (1964) and its later amendments.

### Study design

A qualitative interview-study was performed to explore patients’ and healthcare providers’ perspectives on relevant PROs and personal, contextual and procedural factors that might affect these outcomes during ILVR. In addition, preferences in implementation of PROMs in ILVR were solicited. Semi-structured interviews were conducted from March to August 2022 using an interview guide specifically designed for this study. Interview guides were designed for patients and healthcare providers (Appendix 1). Relevant PROs and possible influencing personal, contextual and procedural factors were addressed by asking participants about improvement during ILVR. in addition, the determinants proposed by Fleuren [39] were used for suggestions on hypothetical implementation of PROMs (based on the found PROs) in ILVR. These determinants, i.e. innovation characteristics, adopting user, socio-political context, and organization, provide a framework to identify and understand the factors that can influence the implementation of an innovation in healthcare. Although Fleuren’s framework is typically applied to evaluate actual implementations, we used it to explore the future hypothetical implementation of PROMs. This was achieved by asking participants about current methods of measuring progress, exploring how progress measurement should be approached, and by identifying additional relevant factors for implementing PRO(M)s in ILVR.

### Participants

Participants were recruited from the only Dutch organization specialized in providing ILVR, i.e. Royal Dutch Visio, location Het Loo Erf in Apeldoorn. Patients who were currently enrolled in the ILVR program (“current patients”) or completed the ILVR program maximum two years prior to the interview (“former patients”), and healthcare providers working within the ILVR organization were invited to participate. Current and former patients were purposively recruited after program counselors selected potential participants based on eligibility criteria and willingness to participate.

Eligible patients were 18 years and older, were in the ILVR program for at least eight weeks or completed the ILVR program between January 2020 and January 2022. A serious illness or cognitive disability lead to exclusion of patients. Healthcare providers were recruited by sending a digital request to all professional groups involved in ILVR: movement therapy, videology, occupational therapy, computer training, program counselling, creative therapy, braille, music, psychology and language training. The professional groups were asked to nominate one healthcare provider to participate in this study. Eligible healthcare providers had at least five years of experience working with patients. All potential participants received information and consent forms and could contact the research group in case of any questions. Written consent was provided by 30 participants. One former patient could not be reached after consent and did not participate.

### Data collection

Semi-structured interviews with individual participants were conducted by TS (current patients) and EvM (former patients and healthcare providers). Interviews with current patients were held at the ILVR organization, interviews with former patients took place according to their preferences, e.g. at home or via video call, and interviews with healthcare providers were conducted via video call. The mean duration of the interviews was 57 min (range 25 to 96 min). All interviews were digitally recorded and transcribed verbatim by a professional transcription service. TS and EvM reviewed all transcripts for accuracy, paying particular attention to unclear or incomplete sections, which were checked against the audio recordings. Although member checking was not conducted, we remained attentive to context and nuance of participants’ statements during the analyses.

### Analysis

Interview data were analyzed to explore relevant PROs in ILVR, factors affecting improvement during ILVR, and preferences in implementation of PROMs to measure PROs. Coding and analysis were performed by three researchers (EvM, HvdA and TS) using MAXQDA standard 2020 software, version 20.3.0.

The coding process combined a deductive and inductive approach. The deductive framework was based on three main domains of the codebook that were derived from the research questions, i.e (1). relevant Patient-Reported Outcomes (PROs) (2), personal, contextual, and procedural factors affecting ILVR, and (3) suggestions for future implementation of PRO(M)s in ILVR. These domains provided the thematic structure for the analysis. Coding was guided by these main domains, but without the use of predefined codes; instead, specific codes were developed inductively within each domain through a close and systematic examination of the data.

The coding process followed an iterative and collaborative approach, starting with open coding in 6 out of 29 interviews, selecting 2 for each stakeholder group, i.e. current patients, former patients, and healthcare providers. Multiple meetings were held to review, discuss and assign codes from these six interviews to relevant categories. Once consensus was reached, a first version of the codebook was developed. This codebook was then used to code all 29 interviews, including a recoding of the initial six interviews. During the coding process, suggestions arose to create new codes, merge existing ones, or split them into more specific subcodes. These suggestions were discussed within the research team until consensus was reached, and the codebook was updated accordingly. The coding of the final interview did not yield any new codes, indicating that data saturation was likely obtained. After initial coding, codes were grouped into broader themes. This involved an iterative discussion among the researchers, refining and reorganizing codes and subthemes. This iterative process was repeated several times, resulting in the final codebook (Appendix 2).

## Results

### Participant characteristics

In total, 29 interviews were performed with current patients, former patients, and healthcare providers. Eleven current patients and eight former patients participated in the study. Patients ranged in age between 19 and 67 years, with a mean age of 50. Patients reported various eye conditions as cause of their vision loss. An overview of patient characteristics is provided in Table 1. In addition, ten female healthcare providers were interviewed, each representing one of the professional groups in the ILVR program. These included two occupational therapists, one music therapist, one psychologist, one creative arts therapist, one program counselor, and four trainers specializing in braille, assistive technology and communication, mobility, or Dutch as a second language. Some of them also had previous experience in other professional roles. On average, the healthcare providers had 16 years of experience working in low vision services, with a range between 7 and 26.5 years. Most of them worked exclusively at the ILVR program throughout their career in low vision services.

**Table 1:** Current and former patients’ characteristics

	Current patients (*n* = 11) Mean (SD) [range]	Former patients (*n* = 8) Mean (SD) [range]
**Age** in years	50.09 (18.67) [19–67]	49.25 (7.38) [37–61]
**Rehabilitation** in months	5.75 (3.78) [2–15]	10.59 (1.75) [8–14]†
**Time since rehabilitation** in months	NA	9 (4.60) [5–18]†
	N (%)	N (%)
**Gender** female	6 (54.55%)	4 (50.00%)
**Vision impairment** ^a^		
Mild vision impairment	3 (27.27%)	1 (12.50%)
Moderate vision impairment	4 (36.36%)	2 (25.00%)
Severe vision impairment	4 (36.36%)	5 (62.50%)
**Cause vision loss** ^b^		
Retinal disease Optic nerve disorder	9 (81.81%) 4 (36.36%)	5 (62.50%) 3 (37.50%)
Refractive errors	2 (18.18%)	2 (25.00%)
Other	2 (18.18%)	1 (12.50%)
**Living situation during ILVR**		
Alone	3 (27.27%)	0 (0.00%)
With partner	4 (36.36%)	2 (25.00%)^c^
With partner and children	2 (18.18%)	6 (75.00%)
With parents and siblings	2 (18.18%)	0 (0.00%)
**Physical comorbidities‡**		
0	5 (45.45%)	7 (87.50%)
1	4 (36.36%)	1 (12.50%)
≥ 2	2 (18.18%)	0 (0.00%)

ILVR inpatient low vision rehabilitation; N number; NA Not applicable; SD Standard deviation

^a^ according to World Health Organization (WHO) criteria

^b^ vision loss was caused by ablatio retinae, acromegaly, cicatricial pemphigoid, cone rod dystrophy, epitheliopathy, fish eye disease, glaucoma, macular degeneration, macular edema, neuromyelitis optica, optic nerve damage following brain tumor, optic atrophy, pseudoxanthoma elasticum, retinal hemorrhage, retinitis pigmentosa, and severe myopia. Some clients fitted in more than one category

^**c**^ one relationship ended during rehabilitation

† Information was missing in one former patient

‡ physical comorbidities included diabetes mellitus, osteoporosis, autoimmune disease, hearing impairment, heart condition and liver failure

### Patient-reported outcomes in ILVR

Participants mentioned several areas in which they progressed during ILVR. These PROs were related to: patients’ mindset, knowledge and strategies, practical skills, self-reliance, social participation, relationships and personal development. An overview of these dimensions and subdimensions is presented in Table 2. Most topics were reported by both stakeholder groups, except for learning to do things in other ways, information and communication strategies, administration and finance, leisure, family life, and peer support (patients only), and personal care and trust (healthcare providers only).

**Table 2:** Overview of dimensions and subdimensions of patient-reported outcomes in inpatient low vision rehabilitation

Dimension	Sub dimensions	Reported by
Mindset	• Grief • Openness about vision impairment • Focus on possibilities • Vision impairment as part of identity • Meaning of life	• Patients and healthcare providers • Patients and healthcare providers • Patients and healthcare providers • Patients and healthcare providers • Patients and healthcare providers
Knowledge and strategies	• Knowledge of vision impairment • Energy balance • Learning to do things in other ways • Non-visual work • Willingness to use aids • Information and communication strategies	• Patients and healthcare providers • Patients and healthcare providers • Patients • Patients and healthcare providers • Patients and healthcare providers • Patients
Practical skills	• Personal care • Household • Administration and finance • Leisure • Mobility • Use of technology • Braille	• Healthcare providers • Patients and healthcare providers • Patients • Patients • Patients and healthcare providers • Patients and healthcare providers • Patients and healthcare providers
Self-reliance	• Independency • Assertiveness • Courage • Practice and use at home	• Patients and healthcare providers • Patients and healthcare providers • Patients and healthcare providers • Patients and healthcare providers
Social participation	• Participation strategies • Work • Daily activities • Language skills and integration	• Patients and healthcare providers • Patients and healthcare providers • Patients and healthcare providers • Patients and healthcare providers
Relationships	• Social skills • Family life • Peer support • Trust	• Patients and healthcare providers • Patients • Patients • Healthcare providers
Personal development	• Self-esteem • Self-confidence • Optimism towards the future • Self-understanding • Self-compassion	• Patients and healthcare providers • Patients and healthcare providers • Patients and healthcare providers • Patients and healthcare providers • Patients and healthcare providers

#### Mindset

Half of the participants mentioned that patients became more aware of their VI, and less often experienced inner struggles associated with having VI and its limiting consequences in daily life. Before ILVR, some patients kept their VI hidden, but during the ILVR, they learned to be open about it, discuss their experiences with loved ones, and manage the pain that may arise during these conversations. It improved patients’ self-image, and patients ‘entered a new life and left the old life behind’, which helped them to identify opportunities and solutions to challenges, and to pursue purpose and personal values.

*Thanks to my growth here*,* I dare to share my VI with my social environment. I hid it very well through excuses and tricks. I did not always realize I was doing that. It just happened automatically. A lot of people did not know I had a VI. Since I started ILVR I find it easier to tell my environment I have a VI. That really feels like a burden off my shoulders. I no longer have to make up excuses.* (Current patient, 35 years, moderate VI)

#### Knowledge and strategies

Mainly patients mentioned they realized their VI was more severe and had more impact on their daily life than they initially thought, and helped them to better understand their needs and capabilities. They also learned about how (their own) energy balance works, such as learning to recognize fatigue, understanding the impact of having VI, and how to maintain balance by recognizing and setting boundaries, using aids, working more non-visually, accepting help, and managing energy levels.

On a practical level, patients indicated they learned to perform tasks differently and discovered new ways of communicating. They gave examples of working in a structured way by dividing plans in several steps, by rearranging their perception strategies through the use of other senses rather than their residual vision, and by replacing non-verbal cues with auditory or tactile cues.

*I did not know how to work with a computer while having a VI. I did not have speech software. When I came here*,* I discovered speech software. Now I can just read my e-mail*,* go on the internet*,* search for things*,* news*,* banking. I can really do a lot without seeing. […] Sometimes when I am on the computer it seems like I do not have a VI. I forget I do not see. Also when I send messages with my mobile phone or need to make a phone call.* (Current patient, 37 years, moderate VI)

#### Practical skills

Patients and healthcare providers indicated patients relearned to perform self-care, household chores, grocery shopping, cooking and handling administrative and financial responsibilities, such as withdrawing money and making (online) payments, and reinvented sports, exercise, and creative outlets like music, art and woodworking, which served as a form of relaxation, and emotional expression and processing. Patients and healthcare providers also noted improvements in mobility skills, enabling patients to orientate and navigate independently, confidently using aids, and utilizing public transport.

*Crossing the streets was scary*,* because I did not see. Now I have learned to cross the streets with the white cane and to listen to traffic. At home*,* I close my eyes and cross the streets. Seriously*,* I do not bother to look anymore. Eyes closed and listening is so much easier on the eyes. And I just have to rely on that.* (Current patient, 66 years, mild VI)

Improved technological skills were mentioned frequently, which included proficient use of mobile phones and computers. Patients became skilled at sending messages, browsing websites, and utilizing apps, voice-over features, and hotkeys. In addition, several patients and healthcare providers mentioned the mastery of braille and its applications in daily life.

#### Self-reliance

Most participants emphasized the importance of the patients’ (sense of) independence, both in managing their daily lives and in embracing the need for occasional support in which asking for help can be a strength and does not undermine their self-esteem. They also reported increased assertiveness, openness and resilience, and ultimately indicated active practicing and integrating everything they have learned into their daily lives fostered self-reliance. Also increased assertiveness, openness and resilience

*Getting over that you have to ask other people for help and that it helps you to show you have a disability. Whilst*,* of course*,* it does not feel that way. For example*,* for a lot of people it feels like they show that they are vulnerable when asking for help. But then it finally makes sense: it helps them to show the outside world that they have a disability.* (Healthcare provider, psychologist)

#### Social participation

Progress also included rejoining society, with a focus on developing a participation strategy. This strategy emphasized developing skills and creating plans for employment, pursuing education or training, and engaging in social activities. Participants mentioned the importance of adapting work arrangements, e.g. part-time employment, finding job opportunities, acquiring work-related skills, and exploring volunteer options.

*Well*,* suppose you cannot do your work anymore. Here you get enough tools to go back to your workplace. Or suppose you could not finish your study because you could no longer read all those documents and you could not take your notes anymore. Here you get enough tools to do all these things again.* (Healthcare provider, technology and braille)

#### Relationships

In relationships, participants mentioned acquiring new social skills related to having VI, such as explaining their condition, presenting themselves, ensuring clear communication, and establishing relationships with their potential need for help in mind. Patients emphasized (the need for) attention for family life, e.g. position and role within the family and strengthening family bonds, and many patients noted they maintained permanent contact with fellow patients, expanding their circle of friends. Moreover, patients regained trust in others after recognizing that people were willing to help them, rather than being malicious or making fun of them.

*By getting in contact with peers*,* I have also made new contacts. People in the same situation I can talk to*,* who sometimes give me tips and advice or whom I can help*,* which makes me feel useful in society again.* (Former patient, 50 years, severe VI)

#### Personal development

An increase of self-confidence and self-esteem was often mentioned by both patients and healthcare providers. Patients gained confidence in their abilities through learning practical, social and emotional skills. They felt worthwhile again, had a more positive self-image and no longer felt inferior to others. Some participants indicated patients’ greater optimism in the future, seeing the world as less intimidating.

*Before I started the ILVR program*,* I thought the whole world had collapsed. Almost everything I normally did was no longer possible. I have outdone myself in several areas and it made me feel better.* (Current patient, 66 years, mild VI)

Moreover, patients gained a deeper understanding of their emotions, feelings and thoughts, also in relation to their VI, and learned to manage them more effectively. Some developed more self-compassion by taking into account the impact of their VI, the importance of managing energy, and taking rest.

### Personal, contextual and procedural factors affecting desired rehabilitation outcomes

Personal, contextual and procedural factors affecting desired outcomes during ILVR were identified and categorized in (1) patient characteristics, (2) the patient’s coping mechanism, (3) social support, (4) rehabilitation circumstances, (5) quality of care, and (6) transfer to home. Table 3 represents all hindering (“-”) and enhancing (“+”) factors mentioned by patients and healthcare providers. Most factors were reported by both stakeholder groups, except for physical condition and practice and rehearsal (patients only), and foreign language, self-awareness, quality social network, single provider limits market competition and hospitalization (healthcare providers only).

**Table 3:** Overview of personal, contextual and procedural factors affecting desired outcomes during ILVR

Theme	Personal, contextual and procedural factors	Reported by
1. Patient characteristics	• Age (+,-) • Psychological and psychiatric problems (-) • Physical condition (-) • Cognitive functioning (-) • Foreign language (-) • Loss of energy (-)	• Patients and healthcare providers • Patients and healthcare providers • Patients • Patients and healthcare providers • Healthcare providers • Patients and healthcare providers
2. Coping mechanisms	• Coping strategies (+) • Adaptation to VI (+,-) • Intrinsic motivation (+) • Self-awareness (+) • Dare to be vulnerable (+)	• Patients and healthcare providers • Patients and healthcare providers • Patients and healthcare providers • Healthcare providers • Patients and healthcare providers
3. Social support	• Quality social network (+,-) • Social situation at home (+,-) • Peer support (+,-)	• Healthcare providers • Patients and healthcare providers • Patients and healthcare providers
4. Rehabilitation circumstances	• Major events (-) • Planning and absenteeism (-) • Reassurance to take time to learn (+,-) • Regain stability (+) • Practice and rehearsal (+)	• Patients and healthcare providers • Patients and healthcare providers • Patients and healthcare providers • Patients and healthcare providers • Patients
5. Quality of care	• Contact between healthcare provider and patient (+) • Attitude healthcare provider (+,-) • Alignment between healthcare providers (+,-) • Supply oriented versus customization (+,-) • Single provider limits market competition (-)	• Patients and healthcare providers • Patients and healthcare providers • Patients and healthcare providers • Patients and healthcare providers • Healthcare providers
6. Transfer to home environment	• Structure and safety (+,-) • Hospitalization (-) • Deployment of regional care (+) • Involving relatives (+)	• Patients and healthcare providers • Healthcare providers • Patients and healthcare providers • Patients and healthcare providers

ILVR inpatient low vision rehabilitation, - negative influence; + positive influence

#### Patient characteristics

Two patients believed younger patients have more potential to reach their desired rehabilitation outcomes as they learn new things faster, while two healthcare providers mentioned that younger patients might struggle more during ILVR due to their inability to process the changes due to their VI and societal role expectations.

*You want to go to work*,* raise your children well*,* bring your children to soccer practice*,* but you are not able do to this on your own. But when you are older*,* your children are grown ups and you have had your career*,* so you do not have to take that into account anymore.*(Healthcare provider, creative therapist)

Additional psychological and psychiatric problems, e.g. mental and emotional vulnerability, developmental-, personality- and mood disorders, and previous trauma, were mentioned as having a negative influence on achieving rehabilitation outcomes. Patients reported that physical illnesses and related complaints, i.e. pain, movement restrictions and lack of energy, hindered them to achieve rehabilitation goals in a timely manner, or required them to adjust their goals.

Other factors that were mentioned to hinder achieving desired outcomes included low energy levels, insufficient Dutch language skills and/or reduced cognitive functioning. Low energy resulted from challenges in managing activity levels, poor sleep or physical illnesses. Problems in cognitive functioning were most often mentioned by healthcare providers giving examples of intellectual disabilities, memory problems and slow information processing.

#### Coping mechanisms

Participants thought patients benefit from being more proactive and flexible in trying new approaches. The patients’ attitude and stage of adaptation to having VI impacts rehabilitation, since effective adaptation and rehabilitation both depend on the ability to approach things differently. Patients with limited awareness or acceptance of having VI may cling to their former selves, are more reluctant to alternatives and assistive aids, and this resistance might lead to obstruction in rehabilitation and consequently worse or delayed positive rehabilitation outcomes.

*Only when you accept things*,* you are ready to do things differently than you are used to.* (Current patient, 35 years, moderate VI)

Moreover, participants mentioned that rehabilitation outcomes are enhanced by intrinsic motivation, e.g. drive to learn, learning to cope with VI, dissatisfaction with one’s current life, and a desire to be self-sufficient. Some healthcare providers pointed out that patients who are self-aware about their values and needs, and who can engage in self-reflection, tend to benefit most from rehabilitation. Many patients developed these qualities during ILVR. Three participants emphasized openness and vulnerability helps in getting to the core of rehabilitation and subsequently receiving adequate support.

#### Social support

Two healthcare providers discussed the influence on rehabilitation outcomes of having a supportive social network of family, friends and colleagues, offering practical and emotional support based on equality. Especially the home situation was mentioned as an important factor. Patients felt receiving support from partners, children and parents to rehabilitate at the institution, and to recover and practice new behavior at home were crucial for success. Lack of this support, excessive responsibilities in taking care of children or partner, pressure from work, and partners or parents who experience difficulties with the changing autonomy of the patients, tend to limit patients in incorporating everything they have learned.

*Well*,* in order to be able to do things yourself and also to be able to apply things at home*,* you also need an environment that cooperates. For example with very young people who still live at home with their parents and do not yet have their own household*,* it is sometimes more difficult. But also*,* partners who have become very accustomed to the fact that the other person can do things less well. Or who are afraid that they cannot do it*,* and therefore find it difficult to give them back their autonomy and just let someone practice and occasionally let things go wrong.* (Healthcare provider, occupational therapist)

Healthcare providers and patients stressed the importance of connecting with others with VI. These interactions contribute to patients’ sense of belonging because they experience a shared process, learn from each other by exchanging experiences and tips, and improve social skills. However, there is a risk these interactions reinforce negative experience and a lack of relaxation during rehabilitation due to intensive contact with other patients.

#### Rehabilitation circumstances

A frequently mentioned negative factor was inefficient use of rehabilitation hours due to poor planning, absenteeism, lack of communication and rescheduling, starting trainings too late, quitting too early, and chitchatting. This led to frustration, decreased motivation and wasted energy in patients and hence, potential negative outcomes.

Major life-events, such as deaths, breakups, legal disputes, medical examinations, upcoming surgeries or unresolved emotional events, distracted patients from reaching rehabilitation goals. Participants mentioned that reassurance to take time for learning and processing is encouraging, as patients often feel rushed and eager to learn everything they can. They need time to accept they cannot and do not have to learn everything, and are allowed to make mistakes without pressure of average processing times and time limits. Practice and repetition were mentioned by patients as important to master new skills. Moreover, several patients appreciated having time to settle or stabilize at the start of rehabilitation, allowing them to calm down and focus on their own needs. One healthcare provider noted that some patients need more time due to the severity or sudden onset of their VI.

*Someone who suddenly becomes visually impaired needs stabilization*,* because that person is completely confused about what happened. You need to stabilize and make sure someone calms down and gets clear their minds*,* because otherwise they cannot learn any new skills or tips. […] Someone who had VI all his life and suffered a burnout or worse*,* should also stabilize first. Though I think this often goes a bit faster*,* since he is identifying more as having VI.* (Healthcare provider, creative therapist)

#### Quality of care

Mainly patients emphasized that contact with trainers and healthcare providers enhanced their rehabilitation results, especially when there was a sense of connection and equality, and when healthcare providers were seen as competent. Both patients and healthcare providers thought the healthcare providers’ attitude affects rehabilitation outcomes. Sincere, enthusiastic healthcare providers who discuss patients’ feelings, answer questions, and propagate and facilitate patients to be in control of their own rehabilitation, enhanced improvement during rehabilitation.

*I think it is very important how you approach a patient during conversation*,* so let the patient be in control. A healthcare provider can coach a patient*,* but patients have to initiate behavioral change themselves. I noticed that whenever I impose something*,* it just does not work.* (Healthcare provider, occupational therapist)

Patients often encountered changes in trainers and noticed differences in their knowledge, approach and advice, which to some degree made patients feel they had to start over with each new trainer. Healthcare providers and patients mentioned that consistent approaches and information among healthcare providers contributed to take over each other’s work. Moreover, use of multidisciplinary discussions to address stagnations strengthens rehabilitation.

*If we teach braille to one patient with three colleagues*,* and I tell him A*,* and my colleagues tell him B*,* it is very annoying for the patient. It can cause irritation and tension*,* and does not work. We should say the same things and be aligned*,* also between different departments.* (Healthcare provider, language and braille trainer)

One healthcare provider expressed her desire to develop more tailored rehabilitation plans to minimize unnecessary energy losses in patients, and better address patients’ needs. Another healthcare provider mentioned the current use of a questionnaire to get insight into training methods (visual versus non-visual) and noted: “*Use of this questionnaire is an improvement*,* since sometimes it is a long journey to find out the right training method. Previously it became clear at the end of the rehabilitation program. And now we have an answer much faster*,* and therefore we can help a patient much better.”* (Healthcare provider, occupational therapist).

#### Transfer to home environment

Both patients and healthcare providers stated that the structure and sense of safety provided during ILVR, helps patients to maintain focus on their goals. However, this structure and safety often fades when patients complete rehabilitation, leading to patients feeling anxious to returning home. One healthcare provider mentioned the risk of hospitalization:

*People who are better off at ILVR than at home*,* who benefit from not achieving their goals*,* hoping they can extend their rehabilitation and postpone the problem of going home. For example*,* lonely patients*,* who only have to walk down for a chat*,* a hand on their shoulder to wish them the best*,* a shoulder to cry on. Everything is within reach. Then that black hole at the end of rehabilitation patients sometimes talk about*,* gets real and bigger.* (Healthcare provider, psychomotor therapist)

Participants mentioned that a graduate transition at the end of rehabilitation, involving relatives and regional care, supports an effective transfer from rehabilitation to home. Time spent at home during rehabilitation, as seen during COVID-19, helped patients practice skills, apply knowledge, and identify remaining or new rehabilitation goals.

### Implementation preferences regarding patient-reported outcome measures

#### Development PROMs

Patients and healthcare providers expressed preferences for monitoring progress during ILVR by using both quantitative and qualitative measurement methods. Quantitative methods, like scales and (short) questionnaires, were valued for their objectivity, measurability, presence of criteria, and ability to gain insight into patient’s level of progress. These methods were seen as helpful to not forget things and avoid patient’s dependency on healthcare providers, but were critiqued for potential misinterpretation, performance pressure, complexity, lack of nuance, and reliability of these ‘snapshots’. Qualitative methods, including (non-)structured conversations and interviews, and observations lists, could help to get additional information and were seen as less theoretical and easier for patients, but also time- and energy consuming. Some participants suggested a combined approach by structuring the interview based on a questionnaire, or administering a questionnaire followed by a discussion in which patients can elaborate on their responses.

Various thoughts on patients’ rehabilitation goals and development of the instrument were shared. Some participants mentioned that rehabilitation goals change during ILVR due to quickly setting goals at the start or gaining new insights during ILVR. Moreover, some discussed a differentiation in PROs and PROMs, reflecting a certain variety of rehabilitation goals between patients, while others thought the instrument could be uniform, suggesting that all patients ultimately aim for similar overarching goals during ILVR.

Participants suggested two options in terms of frequency of applying PROMs: either a fixed interval of several weeks (suggestions from 3 to 12 weeks), or at key stages during ILVR (i.e. intake, midway, discharge and follow-up) to avoid an excessive frequency and therefore time-consuming task in longer ILVR programs. Some participants specifically suggested follow-up administration to gain insight into long-term effectiveness of rehabilitation to map its durability, and to improve future programs.

Most participants thought that patients should assess their progress themselves, or together with trainers. In self-assessment, participants emphasized the importance of taking the diversity in needs and limitations of the target group into account by designing user-friendly and accessible instruments.

*You have to take into account our patients having VI. So questionnaires can quickly become a burden*,* also digitally I think. Especially at the very beginning*,* you cannot really ask that of them. I think it should be administered during a conversation. For example*,* the counselor goes through the questionnaire with the patient. Or the patient has to say he can do it himself.* (Healthcare provider, occupational therapist)

Participants mentioned several healthcare providers that could be involved in applying the instrument, such as the patient’s program counselor, trainers, or less involved or even external professionals. They expected program counselors to be able to perform such measurements, due to their involvement, skills and bond with the patient. Involved trainers were suggested since they are able to weigh and calibrate the outcomes. On the other hand, some participants expressed concerns about potential patient dependency of program counselors and a risk of patients glorifying the scores, and advocated for more independent assessors. Considering sharing the results, patients preferred their program counselor or trainer to either discuss the results in a personal conversation or during a multidisciplinary meeting. Two patients suggested the use of percentages to show progress.

#### Implementation in clinical practice

Most participants expected an added value in using PROMs, since it helps to assess the current state of a patient’s rehabilitation and goals, and whether adjustment or expansion of the ILVR is desirable. Some participants also valued the opportunity to gain insight into the overall ILVR program. These insights could help to compare individual to average rehabilitation scores, identify generic stagnations during the rehabilitation process, and provide suggestions for adjustment, which supports ILVR effectiveness. A few current patients were satisfied with current progress measurement methods.

Participants proposed integrating the instrument’s administration and results into existing meetings with trainers, the program counselor or the multidisciplinary team. Participants discussed the varying levels patient and trainer attendance during multidisciplinary meetings and difficulties in determination of progress. They also highlighted the importance of establishing shared goals across departments, and fostering deeper discussions during these meetings.

*I think the multidisciplinary meetings in which we reflect on the patient’s bigger picture are very pleasant. I hate the multidisciplinary meetings where everyone just reads their report out loud. That has less added value for me*,* because I could have read that myself. I think that we should discuss the part that is not written down.* (Healthcare provider, psychomotor therapist)

Other preconditions mentioned by participants were time and information. They underlined the impact of repeated measurements on effective rehabilitation time, and they advocated for a user-friendly and concise instrument, possibly administered outside training hours and/or by external administrators. Healthcare providers highlighted the need for research outcomes, information about the benefits of using the instrument for patients and healthcare providers, and a uniform procedure for effective implementation.

## Discussion

This study was performed as a first step in development of a set of PROMs to measure the (cost-)effectiveness of ILVR. The aim of this study was to explore relevant PROs in ILVR, contextual and procedural factors affecting the desired rehabilitation outcomes, and PROM implementation in an ILVR setting.

Relevant PROs that were found were categorized into seven dimensions, i.e. mindset, knowledge and strategies, practical skills, self-reliance, social participation, relationships and personal development. According to previous reviews on measuring effects of low vision rehabilitation, numerous outcomes have been used to assess its effectiveness, e.g. activities in daily living (ability, dependence and confidence), mobility, reading, use of low vision aids, self-esteem and efficacy, general and vision related quality of life, mental health, and participation [7, 10, 24, 40, 41]. Similarities between these outcomes and the seven domains of relevant ILVR outcomes identified in this study can be found in measuring patient’s practical skills, personal development and to some extent in knowledge and strategies, and social participation. However, ILVR seems to foster rehabilitation in additional dimensions, such as the patient’s mindset and self-reliance. The additional progress on these dimensions might be due to a different and more intensive type of rehabilitation, which possibly provides more space for these aspects. The PROs and subsequently PROMs used in ILVR should be different from and more extensive than those used in regular LVR.

In order to operationalize the relevant PROs into PROMs, the “User’s guide to implementing Patient reported outcomes assessment in clinical practice” can be used [42]. This guide is designed to support clinicians in integrating PROMs into clinical practice to enhance patient management and care by describing nine relevant questions to consider when implementing PROMs. It suggests for example to consider both generic and disease-specific questionnaires, both having specific advantages and disadvantages [43]. It seems the generic PROMs from the Patient-Reported Outcome Measurement Information System (PROMIS), which are used by many researchers, largely meet the need for measuring effectiveness in ILVR programs, since they provide PROMs to address physical, mental and social health [44]. In addition, condition-specific PROMs (i.e. vision impairment or specific eye disorders) are available to measure visual functioning, participation and quality of life in individuals with VI [45–49]. Advantages and disadvantages of using these condition-specific measures over generic PROMs should be weighed, and if a vision-specific PROM is preferred a well-considered decision should be made between the variety of vision-specific measures [7, 22]. Some relevant ILVR outcomes seem to be vision-specific and are not captured in generic PROMs. For example aspects from the patient’s mindset, knowledge and use of strategies, that might reflect their adaptation to having VI, could be measured with the Adaptation to Vision Loss scale [49]. 

Personal, contextual and procedural factors affecting desirable outcomes during ILVR were identified at the level of the patient (i.e. characteristics, coping mechanisms, and social support), and the organization (i.e. rehabilitation circumstances, quality of care, and transfer to the home environment). Several of these patient factors, such as age, level of education, physical and mental health, personality traits, confidence, knowledge about low vision, and motivation are associated with better reading outcomes, durable use of low vision aids and adaptation to vision loss [45, 50–52], and seem to be important in ILVR as well. Vision related factors were not explicitly mentioned as potential factors affecting rehabilitation outcomes, but diagnosis, visual acuity, residual vision and changes in vision seem to be associated with visual ability, reading ability and use of low vision aids [32, 33, 50]. Understanding the predictive value of patient characteristics can help to identify patients who are will likely to benefit from the ILVR program and those for whom ILVR does not seem feasible. It can also determine patients who may require specific support to enhance the effectiveness of their individual ILVR. Predictive factors at organization level could provide suggestions to improve quality of care leading to more effective ILVR programs, e.g. uniformity of care since consistent training is associated with durable use of low vision aids [51]. 

The identified PROs and personal, contextual and procedural factors affecting these PROs can be used to design the infrastructure to use PROMs into clinical practice. It is important to address the feasibility and acceptability of these PROMS in ILVR programs in both patients and healthcare providers. In general, healthcare providers and patients were positive about implementing PROMs, but accessibility and the burden for patients and healthcare providers should be taken into account. The preferred mode of completing PROMs in our study was by patients themselves, either by using paper forms, interviews or digital questionnaires. However, in line with previous studies [53], individuals with VI can struggle with completing questionnaires themselves on paper or digitally, especially at the start of ILVR. PROMs could therefore be administered through a telephone interactive voice response system [54], or as suggested by participants, assisted by a professional. An independent (third-party) professional can help to minimize the risk of patients reporting overly positive answers. This issue is particularly notable among patients receiving long-term rehabilitation when assessed by a healthcare provider who is directly involved in their care [10]. In addition, the frequency of completing PROMs should be considered. Participants most often suggested to administer the PROMs every six weeks and to discuss the outcomes before and during the six-weekly multidisciplinary meetings. A high frequency of administering and scoring PROMs could be a burden to patients [42]. Especially, since individuals with VI often experience fatigue [55]. Therefore, the number of measures in the PROM set and its length should be limited. Computer adaptive test (CAT) measures have the potential to decrease the burden of questionnaire administration in patients and clinicians [56], and are compatible with individuals with VI [57]. The advantages of CAT measures encourages the use of generic PROMs, for instance incorporated in PROMIS, since many of these PROMs already are computer adaptive [58]. Another approach to decrease the measurement’s burden, is to distinguish between PROMs used to adjust rehabilitation goals during multidisciplinary meetings (every six weeks), and PROMs that only need to be completed at certain points of ILVR, e.g. start, midway, end and follow-up.

### Strengths and limitations

This study explored relevant PROs, potential factors influencing progress during ILVR, and implementation of PROMs in ILVR. Exploration from the perspective of current patients, former patients and healthcare providers working at IVLR contributed to valuable data from a broad perspective. These different perspectives complement each other, as current patients may struggle to anticipate the needs after ILVR and are dependent on the ILVR organization, while former patients might experience recall bias [59] making it difficult for them to remember what they considered important during ILVR. The heterogeneity of patients regarding age, VI, comorbid conditions, family situation, work-life, and cultural background, further contributed to a comprehensive overview of PROs and the potential influences on rehabilitation outcomes. Moreover, including the healthcare providers’ perspective adds to the patients’ individual experiences by offering a general view across ILVR patients.

Although Fleuren’s framework is typically applied to evaluate the implementation of existing innovations, we consider the early consideration of implementation factors during the exploration phase of a PROM set for ILVR as a strength of our study. In our view, this anticipatory approach is valuable, as implementation barriers are often overlooked during innovation development, potentially hindering later uptake and integration into practice. However, since this approach deviates from the original use of the framework, the comparability of our findings with studies that apply this framework strictly at the implementation stage may be limited.

Despite the efforts taken to include a heterogeneous group of patients, patients who do not speak Dutch fluently, have cognitive impairments or intellectual disabilities, were not interviewed. These patients might have different goals during ILVR or experience different factors hindering of enhancing their rehabilitation. The interviews were conducted during the COVID-19 pandemic, which slowed down the data collection process but did not comprise data quality. In fact, it offered additional insights in improving ILVR. By asking participants about what they felt was missing during ILVR, we gained valuable insight into important PRO(M)s. Formal member checking was not conducted, with the risk of misinterpretation or incomplete understanding of the participants’ intended meanings. However, the collaboration between two low vision researchers and one healthcare professional during data analysis, helped to balance contextual insight with critical reflection.

### Implications for clinical practice and future research

Implementing PROMs can reliably examine (cost)-effectiveness of ILVR in general, and can help healthcare providers discuss individual progress in a timely manner. It allows for better determination of when patients have reached their potential for each outcome, enabling adjustments to the rehabilitation process. This study provides insights that can serve as a foundation for further steps toward the use of PROMs in ILVR, particularly in selecting a set of PROs and determining how and when a PROM set should be administered. The operationalization of PROs into PROMs can be supported by a systematic literature review to identify relevant questionnaires based on their content and psychometric properties. Moreover, the mode of administration and potential burden on both patients and healthcare providers should be carefully considered when selecting and defining the set of PROMs, especially given the risk of patient fatigue and limited technological proficiency.

Once the tailored set of PROMs is established, it can be used in a pilot study to investigate the (cost)-effectiveness of ILVR trajectories. In addition, the influence of the personal, contextual and procedural factors on (cost-)effectiveness can be explored. This will help to determine whether patients with specific characteristics require targeted support to improve outcomes, or if particular rehabilitation settings need adjustments to optimize effectiveness.

## Conclusion

Relevant PROs in ILVR, personal, contextual and procedural factors affecting these PROs, and implementation preferences for PROMs in ILVR were explored. Progression in ILVR is expected in various domains and might be affected by several factors in patients and the ILVR itself, e.g. coping mechanisms and rehabilitation circumstances. It seems patients’ progress during ILVR can be monitored by using a combination of generic PROMs and vision-specific PROMs. In developing the actual measurement instrument, the specific needs of both patients and healthcare providers in ILVR should be taken into account, leading to an efficient, accessible and short measurement instrument to monitor progression during and after ILVR. Additional research and consensus between experts in the field is required to establish a tailored set of PROMs for ILVR.

## Supplementary Information

Below is the link to the electronic supplementary material.

Supplementary Material 1

Supplementary Material 2

## Data Availability

The datasets used and/or analysed during the current study are available from the corresponding author on reasonable request.

## Abbreviations

ILVR: Inpatient low vision rehabilitation
LVR: Low vision rehabilitation
NA: Not applicable
PRO: Patient-reported outcome
PROM: Patient-reported outcome measure
SD: Standard deviation
VI: Vision impairment
WHO: World health organization
