# Supplementary material for: Exploring patient-reported outcomes to assess progress in inpatient low vision rehabilitation
Source: J Patient Rep Outcomes. 2025 Nov 18;9:133. doi: 10.1186/s41687-025-00960-8 (PMC12627308; doi:10.1186/s41687-025-00960-8)
Supplement: Supplementary file 1 — Supplementary Material 1 [file 41687_2025_960_MOESM1_ESM.docx]

# Appendix 1: Interview guide patients and healthcare providers

*In this interview guide, the term 'measurement of progression' is used to introduce patient-reported outcomes, as it speaks more directly to the imagination and facilitates clearer interpretation for patients and healthcare providers who may not be familiar with patient-reported outcome (measures).*

| **Topic** | **Questions** |
| --- | --- |
| **Participant characteristics** | Patients   - Can you tell me something about yourself (age, living situation, etc) - What eye problems do you experience at the moment? What is your diagnosis? - Do you also experience other physical and/or psychological problems at the moment? If so, which problems do you experience?  *If former patient*: did you experience these problems also during inpatient low vision rehabilitation? - Do you receive support or treatment for these physical and/or psychological problems, if so in what manner?  *If former patient*: did you receive support or treatment during inpatient low vision rehabilitation?   Healthcare providers   - Can you tell me something about your current profession and responsibilities? - How many years have you been working in the low vision services, and specifically within inpatient low vision rehabilitation? *Was this always in the same profession?* - What does your patient contact look like? - What is / was your role during eligibility assessment and measurement of progression during inpatient low vision rehabilitation in patients? |
| **Added value and feasibility of PRO(M)s*** | Patients   - What do you think of the current way eligibility assessment and measurement of progression during inpatient low vision rehabilitation are performed? *Can you explain your positive and/or negative experiences?* - What do you think are the (dis)advantages of measuring progression?   Healthcare providers   - What do you think of the current way eligibility assessment and measurement of progression during inpatient low vision rehabilitation are performed? *Can you explain your positive and/or negative experiences?* - How do patients respond to eligibility assessment and measurement of progression? *Can you explain your answer, including potential (dis)advantages patients* - What do other healthcare providers within the organization think about current eligibility assessment and measurement of progression? |
| **Progress / Patient-reported outcomes** | Patients   - In what different ways and areas do/did you notice that you are/were progressing during the inpatient low vision rehabilitation?   *Prompt: other area(s)? Or if needed a more specific prompt: what do you think of your progress in for example contact with others, participation in society, personal development?*   - Are there areas that you have not mentioned yet, but where you would (have) like(d) to make progress? If so, which areas? - Which of the areas you mentioned do you consider the most important as a measure of progression and should be included in progress measurements? *This can be multiple areas and explanation is requested.*   Healthcare providers   - What are the areas in which patients can progress during their inpatient low vision rehabilitation?  *Prompt: In which other areas could patients progress? Or if needed a more specific prompt: what do you think of progress for example contact with other, participation in society, personal development.* - Which of the areas you mentioned do you consider the most important as a measure of progression and should be included in progress measurements? *This can be multiple areas and explanation is requested.* |
| **Personal, contextual and procedural factors** | Patients   - Which factors or circumstances made progression **more difficult** for you? *Explanation. Prompts: patient level, professional level, rehabilitation trajectory and organization* - Which factors or circumstances **enhanced** progression for you? *Explanation. Prompts: patient level, professional level, rehabilitation trajectory and organization*   Healthcare providers   - Which factors or circumstances (positive and negative) influences progression in patients? *Prompts: patient level, professional level, rehabilitation trajectory and organization* |
| **Development PROMs*** | Patients   - What is the best way to conduct the measurement of progression? *Prompts: when, how often, by whom, in what way, etc.* - How would you like to receive the results of the measurement of progression? *Prompts: when, how often, by whom, how, who has access/receives results* - Are there any other things to consider when developing or using measurements of progression? *Prompt: things you do not want to happen?*   Healthcare providers   - What is the best way to conduct the measurement of progression? *Prompts: when, how often, by whom, in what way, etc.* - What should be done with the results of the measurement of progression, and what should these results look like? *Prompts: report, feedback, access* |
| **Implementation in clinical practice*** | Healthcare providers   - What do you and your colleagues need to use measurement of progression? *Prompts: education, digital skills, ICT, time, budget, etc.* - How could measurement of progression be used within existing procedures? Is adaptation needed? *Explanation.* - How can we optimize the embedding of measurement of progression? - Which professionals should be involved during implementation? - Are there any other factors on organizational level, so within your organization or department, that play a role in applying this instrument? *Prompt: policy, support from supervisor/management, support colleagues, available expertise, not part of responsibilities, collaboration with other departments, finances, other priorities? Explanation.* - Are there any other factors in the broader context? *Prompt: health insurer, affordability rehabilitation care, etc. Explanation.* - Are there any other factors that can play a role during implementation? *Explanation.* |

PRO(M) patient-reported outcome (measure)
* Exploration of the future hypothetical implementation of PROMS in inpatient low vision rehabilitation based on the Fleuren Framework.^1^

^\^1. Fleuren M, Wiefferink K, Paulussen T (2004) Determinants of innovation within health care organizations: literature review and Delphi study. Int J Qual Health Care.16(2):107-23.
