# Supplementary material for: Exploring patient-reported outcomes to assess progress in inpatient low vision rehabilitation
Source: J Patient Rep Outcomes. 2025 Nov 18;9:133. doi: 10.1186/s41687-025-00960-8 (PMC12627308; doi:10.1186/s41687-025-00960-8)
Supplement: Supplementary file 2 — Supplementary Material 2 [file 41687_2025_960_MOESM2_ESM.docx]

# Codebook *“Exploring Patient-Reported Outcomes to Assess Progress in Inpatient Low Vision Rehabilitation”*

**This codebook consists all codes related to the relevant patient-reported outcomes, personal, contextual and procedural factors affecting progress, and implementation of PRO(M)s within inpatient low vision rehabilitation (ILVR). In this codebook, no distinction is made between statements made by patients or healthcare providers.**

| **1 Relevant patient-reported outcomes** All codes related to the content of patient-reported outcome (measurements) during intensive low vision rehabilitation. | | |
| --- | --- | --- |
| **Dimension** | **Subdimension  (Code in MAXQDA)** | **Description of the code** |
| Mindset | Grief | Everything related to raising awareness of, and reducing internal resistance to, as well as 'accepting' the vision impairment and its associated limitations. |
| Mindset | Openness about vision impairment | Everything related to the idea that conversations about the vision impairment are allowed, and that patients are more open about it with others and no longer keep it hidden. |
| Mindset | Focus on possibilities | Everything related to patients (re)gaining the belief that there are possibilities despite the vision impairment, that they also see solutions, and accept that sometimes things take more time, which in turn expands their world and helps them feel better. |
| Mindset | Vision impairment as part of identity | Everything related to adjusting one's self-image in relation to the vision impairment, embarking on a 'new life' and leaving the 'old' behind, and being able to relate to this change in identity (no longer denying it and adapting to/living according to it). |
| Mindset | Meaning of life | Everything related to (the search for) meaning that aligns with the (new identity), consisting of insight into what patients find valuable in life (which can then lead to a participation strategy). |
| Knowledge and strategies | Knowledge of vision impairment | Everything related to patients gaining insight into their vision impairment, such as visual functions (often thinking they could see more) and the impact the vision impairment has on daily life, which helps them understand the (im)possibilities and needs. |
| Knowledge and strategies | Energy balance | Everything related to insight into (one's own) energy balance, including recognizing fatigue, the influence of the vision impairment, awareness and balance of energy drainers and givers, and learning to set boundaries by using assistive devices, non-visual work, self-care, energy management, and organizing participation. |
| Knowledge and strategies | Learning to do things in other ways | Everything related to patients learning to perform tasks in a different way due to the vision impairment, such as adapting perception, using other senses, and creating a step-by-step plan in advance. |
| Knowledge and strategies | Non-visual work | Everything related to patients learning to work consciously in a non-visual way, such as using speech functions (voice-over), more consciously utilizing touch, and working in a structured manner, where they experience that this is less exhausting than working visually. |
| Knowledge and strategies | Willingsness to use aids | Everything related to getting to know suitable assistive devices and being able and willing to use them with confidence, where patients no longer see assistive devices as a burden but as a helpful tool, are satisfied with their use, and can make choices regarding ease of use and necessity. |
| Knowledge and strategies | Information and communication strategies | Everything related to developing skills and using assistive devices to regain access to information, and finding renewed ways of communication (auditory and digital), which contributes to expanding the patients' world. |
| Practical skills | Personal care | Everything related to performing personal care, such as dressing and self-care. |
| Practical skills | Household | Everything related to being able to (again) perform household tasks independently, such as cleaning and organizing, grocery shopping, and preparing meals. |
| Practical skills | Administration and finance | Everything related to performing administrative tasks and managing finances, such as online banking, withdrawing money, and making card payments. |
| Practical skills | Leisure | Everything related to (re)learning to practice sports and finding (renewed) ways to express themselves creatively (music/art/woodworking). |
| Practical skills | Mobility | Everything related to patients being able to move independently and with more confidence from one place to another, by using assistive devices, better orienting themselves both indoors and outdoors, and being able to use public transport. |
| Practical skills | Use of technology | Everything related to using technical assistive devices and learned skills more (easily), such as apps, sending messages, using shortcuts, voice-over, and visiting websites. |
| Practical skills | Braille | Everything related to learning Braille and its applications in daily life. |
| Self-reliance | Independency | Everything related to the importance of (the feeling of) independence for patients to create their lives autonomously (taking care of themselves, going outside), but also the ability to tolerate dependence (understanding that asking for help can be supportive and does not diminish self-worth). |
| Self-reliance | Assertiveness | Everything related to patients becoming more open and willing to ask for help, expressed through more communication, awareness of their own opinions, choices, and boundaries, and being able to express them. This also includes becoming more resilient, no longer wanting to do everything on their own, not waiting passively, and asking for help. |
| Self-reliance | Courage | Everything related to patients daring to take on (new) things again, and not avoiding challenges or learning processes or leaving them to others. |
| Self-reliance | Practice and use at home | Everything related to the idea that progress for patients means applying what they have learned at intensive low vision rehabilitation in their home environment. |
| Social participation | Participation strategies | Everything related to developing skills and creating plans to re-engage in work, education, courses, social activities, and/or generally having a role in society. |
| Social participation | Work | Everything related to focusing on work and the associated changes, such as working part-time, finding a (different) job, developing skills, and doing volunteer work. |
| Social participation | Daily activities | Everything related to (re)establishing a daily and weekly routine and finding a way to structure the day, including pursuing hobbies and finding relaxation (e.g., through sports and creative activities). |
| Social participation | Language skills and integration | Everything related to non-native patients becoming proficient in the Dutch language and becoming acquainted with society. |
| Relationships | Social skills | Everything related to using social skills in relation to the vision impairment, such as providing explanations, receiving understanding, positioning/presenting oneself, and building relationships. |
| Relationships | Family life | Everything related to the development of patients' roles and positions within their families and strengthening family relationships. |
| Relationships | Peer support | Everything related to maintaining contact with peers, through which patients have expanded their circle of friends and gained satisfaction from exchanging tips and advice on managing their vision impairment. |
| Relationships | Trust | Everything related to having trust in others, believing that they are willing to help, are not malicious, will not ridicule them, and that this is also true at ILVR. |
| Personal development | Self-esteem | Everything related to patients feeling worthwhile again, having a more positive self-image, and not feeling inferior to others. |
| Personal development | Self-confidence | Everything related to the increased self-confidence in patients' abilities, in which the reasons for this increased self-confidence can vary (practical, social, or emotional skills they have learned). |
| Personal development | Optimism towards the future | Everything related to patients (re)gaining trust in the future: the world is not as scary and threatening, and there is hope and confidence that they can influence it. |
| Personal development | Self-understanding | Everything related to patients gaining insight into their own thoughts and emotions (whether or not in relation to the vision impairment) and learning how to cope with them. |
| Personal development | Selfcompassion | Everything related to the development of self-compassion, taking into account the impact of the vision impairment and allowing time for oneself/rest. |
| **2** **Personal, contextual and procedural factors affecting ILVR** Any factors affecting patients’ progress during inpatient low vision rehabilitation resulting in acceleration, deceleration or stagnation of the rehabilitation.  *Factors with negative effect are marked with (-) and factors with positive effect are marked with (+)* | | |
| **Theme** | **Negative effect (-) / Positive effect (+) (Code in MAXQDA)** | **Description of the code** |
| Patient characteristics | Age (+,-) | Everything related to the influence of age on the rehabilitation process, where being young can be a barrier because younger patients may be less advanced in acceptance and may have a greater need to establish a social role in society compared to older individuals. On the other hand, older age is associated with needing to unlearn certain things before being able to learn something new. |
| Patient characteristics | Psychological and psychiatric problems (-) | Everything related to the influence of comorbid mental or psychiatric issues, such as mental and emotional vulnerability (reduced assertiveness), developmental disorders, personality disorders, mood problems, and trauma, where the need for support from mental healthcare services may slow down the rehabilitation process. Good mental health, on the other hand, has a positive impact. |
| Patient characteristics | Physical condition (-) | Everything related to the negative influence of physical conditions and associated complaints, such as pain, motor limitations, and limited energy, resulting in goals not being achieved or needing to be adjusted, or slowing down the rehabilitation process. |
| Patient characteristics | Cognitive functioning (+/-) | Everything related to the influence of cognitive functioning on learning ability, such as being highly intelligent, having a (mild) intellectual disability, memory problems, slower information processing, and the inability to retain what has been learned. |
| Patient characteristics | Foreign language (-) | Everything related to the influence of insufficient mastery of the Dutch language, for example, in applying what has been learned in practice. |
| Patient characteristics | Loss of energy (-) | Everything related to the influence of limited energy levels, imbalance in energy, and lack of rest/sleep during the start and throughout the rehabilitation process. |
| Coping mechanisms | Coping strategies (+/-) | Everything related to the influence of different coping styles, where some mechanisms are unhelpful (e.g., avoidance) and others are helpful (e.g., solution-focused thinking, flexibility), but also where previously effective coping mechanisms no longer work due to the vision impairment (e.g., the loss of visual control used to cope with a history of abuse). |
| Coping mechanisms | Adaptation to VI (+/-) | Everything related to the influence of (the stage of) the acceptance process, in which processing and acceptance are necessary to feel the space to do things differently/rehabilitate. Insufficient awareness or acceptance of having vision impairment leads to a desire to maintain things as they were, not being open to alternatives or assistive devices, and seeking resistance, which results in getting stuck in the rehabilitation process. |
| Coping mechanisms | Intrinsic motivation (+) | Everything related to the fact that intrinsic motivation of the rehabilitant has a positive influence on rehabilitation, with various driving forces mentioned, such as dissatisfaction with the current situation, learning to cope with the vision impairment, wanting to be self-sufficient, and wanting to get the most out of it to achieve this (or driven by curiosity). However, motivation can sometimes diminish, for example, due to a lack of future perspectives regarding work and a stronger focus on pushing for a disability allowance. |
| Coping mechanisms | Self-awareness (+) | Everything related to the fact that patients with self-insight benefit during their rehabilitation, as they already know what is important to them, what their values are, and what brings them fulfillment. They can also critically assess themselves, understand what they need, take control of it, and speak up. However, many patients undergo this development during the rehabilitation process. |
| Coping mechanisms | Dare to be vulnerable (+) | Everything related to patients' willingness (or unwillingness) to allow themselves to be vulnerable, which impacts their capacity to reach the core issues and/or receive the appropriate support. |
| Social support | Quality social network (+/-) | Everything related to the quantity and quality of a (supportive) social network (friends, family, work), providing practical and emotional support based on equality. |
| Social support | Social situation at home (+/-) | Everything related to (a lack of) space and support for rehabilitation at ILVR, taking rest at home, and practicing what has been learned at home. This includes, on one hand, support, understanding, and encouragement from partners, children, and parents, but on the other hand, a lack of such support, responsibility for and concerns about children or a partner, pressure from work, or a partner or parent struggling with changes in the patient's autonomy. |
| Social support | Peer support (+/-) | Everything related to the benefits patients gain from peer support, which contributes to a sense of belonging by going through the same transformation process with like-minded individuals, providing space to learn from each other through exchanging tips and experiences, and aiding in social development. On the other hand, there is a risk of reinforcing negative experiences within ILVR and a lack of taking adequate rest. |
| Rehabilitation circumstances | Major events (-) | All significant events that divert the patient's attention/focus away from rehabilitation, such as deaths, relationship breakups/legal disputes, upcoming surgeries or medical exams, as well as past events that require attention again. |
| Rehabilitation circumstances | Planning and absenteeism(-) | Everything related to the inefficient use of rehabilitation hours due to poor planning (too many gaps between sessions or too tight scheduling), absenteeism of healthcare providers and patients, lack of rescheduling and communication, and not fully utilizing the allocated time (starting too late, finishing too early, unnecessary chatting), which can lead to frustration, wasted energy, and a lack of motivation in patients. |
| Rehabilitation circumstances | Reassurance to take time to learn (+/-) | Everything related to the need for patients to have sufficient time and space to learn and process, allowing to start the acceptance process, where not everything must be learned or mastered, the ability to make mistakes, and taking the necessary time to learn. Patients should not be pressured by average durations or limits set by the health insurance provider. Patients often desire to progress too quickly and take on too much. |
| Rehabilitation circumstances | Regain stability (+) | Everything related to a calm environment and the space to stabilize at the beginning of rehabilitation, such as learning to focus on oneself, detaching from home, and finding calm (possibly in relation to the vision impairment), which contributes to starting the rehabilitation process effectively. Some patients require more time for this than others (e.g., due to the progression of the vision impairment). |
| Rehabilitation circumstances | Practice and rehearsal (+) | Everything related to the fact that (long-term) practice and repetition of actions have a positive effect. |
| Quality of care | Contact between healthcare provider and patient (+) | Everything related to the importance of the relationship between healthcare provider and patient, where the connection between both parties and mutual respect are emphasized, along with the positivity, knowledge, and understanding of the healthcare provider. |
| Quality of care | Attitude healthcare provider (+/-) | Everything related to the influence of the interaction between trainers and patients, focusing on the trainer's attitude towards the patient (openness, accessibility, being able to discuss feelings and questions, sincerity, motivational), and the trainer’s communication and attitude that the patient holds control, rather than the trainer filling in for the patient. |
| Quality of care | Alignment between healthcare providers (+/-) | Everything related to the importance of using the same approach and information within (and between) professional groups, allowing for continuity in care and utilizing multidisciplinary coordination to discuss stagnation in rehabilitation, which contributes to a smooth rehabilitation process. However, this is not always the case due to changes in trainers and their available knowledge. |
| Quality of care | Supply oriented versus customatization (+/-) | Everything related to the use of a standardized approach over a tailored model, of which the standardized approach is not the desired option because (1) patients fear missing out on important aspects and unnecessary energy being wasted, or (2) they feel disconnected from the process, where there is also a need for customization based on patients' needs (visual versus non-visual, offering new forms of creativity or "daily activities," fixed and flexible options, and effective use of time for shorter periods). |
| Quality of care | Single provider limits market competition (-) | Everything related to the possibility that changes or evidence-based improvements within ILVR may be minimal, because it is the only place patients can go to (and each professional group is allowed to determine its own approach). |
| Transfer to home environment | Structure and safety (+/-) | Everything related to the structure and safety provided within ILVR, which helps patients focus on their goals, but that this structure and safety often diminish when leaving ILVR, making many patients anxious about returning home. Offering the option to gradually return from ILVR to the home environment is seen as an opportunity to transfer what has been learned to the home situation. Additionally, the experience from the COVID-19 pandemic shows that time spent at home helps to apply what has been learned in practice and identify areas that still need improvement. |
| Transfer to home environment | Institutionalization (-) | Everything related to the risk of the comforting environment at ILVR contributing to the institutionalization of lonely patients, which can lead to a prolonged rehabilitation process. |
| Transfer to home environment | Deployment of regional care (+) | Everything related to the fact that good collaboration with and involvement of regional care/support during and after rehabilitation can contribute to patients applying what has been learned into their own situation, but that this is not always feasible in every region. |
| Transfer to home environment | Involving relatives (+) | Everything related to involving loved ones during rehabilitation, for example through an experience day, which provides more insight and understanding of the patient's situation, positively impacting the social home environment, but where additional attention may still be needed. |
| **3 Implementation of patient reported outcome (measures)**  Any factors related to design and successful future implementation of patient reported outcome (measures) in inpatient low vision rehabilitation.  Fleuren: characteristics of innovation, organisation, adopting user and socio-political context | | |
| **Theme** | **Factor (Code in MAXQDA)** | **Description of the code** |
| Development PROMs | Qualitative measures: (dis)advantages | Everything related to the qualitative measurement of progress during ILVR, including the specific format (interview) and its pros and cons, such as hearing different perspectives and providing nuance, allowing the trainer to assess the patient’s current status, being less theoretical and therefore clearer, easier for the patient, but also requires a lot of energy and time. |
| Development PROMs | Quantitative measures: (dis)advantages | Everything related to the quantitative measurement of progress during ILVR, including the specific formats (use of scales, (short) questionnaires, structured interviews, matrix filling, observation checklists (yes/no)), and the pros and cons, such as objectivity, insight into the patient’s level, structure (ensuring nothing is overlooked), measurability, providing a standard, reducing dependency, but also the potential for differing interpretations of numbers/scales by patients, pressure to perform, doubts about the reliability of snapshots, complexity of scales and percentages, difficulty in adding nuances or receiving patient input, "choice stress," and lack of clarity when working with schemas. |
| Development PROMs | Combination of measurements | Everything related to the need for using different forms of measurement, including suggestions for structuring a conversation based on a questionnaire, or completing a questionnaire supplemented with a discussion to clarify answers. |
| Development PROMs | Planning PROMs | Everything related to the frequency of the measurements, including intervals and specific moments within the rehabilitation process, and how it may depend on the duration of the program, making a certain number of measurements more appropriate to avoid excessive frequency and time intensity. |
| Development PROMs | Assessor PROMs | Everything related to the considerations of who assesses/records the progress (patient and/or trainer) and the reasoning behind it. |
| Development PROMs | Support assessment PROMs | Everything related to who should be involved in supporting the measurement, including statements about who could play a role in this, with reasoning such as the therapeutic relationship, skills, overview, etc. |
| Development PROMs | Need for follow-up measurement | Everything related to the need for insight into the long-term effectiveness of rehabilitation, so that the sustainability of the effect becomes visible (the essence of rehabilitation) and it provides indications for the design of future programs. |
| Development PROMs | Accessibility for patients | Everything related to the need to take the target group into account (the diversity of needs and limitations of), in terms of usability and accessibility, with suggestions for various methods of administration. |
| Development PROMs | Customization versus uniformity | Everything related to the perspective on the content and design regarding customization and uniformity, where some participants suggest that the content of measurement varies per patient due to differing goals, while others argue that there are overarching goals and that the format is also dependent on preferences. |
| Development PROMs | Sharing results | Everything related to the representation of results, including the method of delivery, timing, the parties involved, the recipient of the results, and, if available, the reasoning behind these preferences. |
| Development PROMs | Changing goals | Everything related to setting general goals and specifying or adjusting those goals later during the rehabilitation, due to patients either forgetting them, gaining new insights, a lack of time during observations at the start of rehabilitation, or insufficient information/awareness at the start of the rehabilitation. It also involves cases where certain goals are found to be unachievable, with some participants believing these can easily be adjusted, while others disagree. |
| Implementation | Time | Everything related to the time intensity of measurements, where there is a need for a method that is as accessible as possible and has minimal impact on training time, not being too burdensome for both the healthcare provider and the patient. A suggestion is that the patient can administer the measurement outstide training time, but if a patient cannot do this on their own, time should be allocated for the patient to receive support. |
| Implementation | Possibility of integration in current processes | Everything related to the ease of integrating the measurement into the current processes at ILVR, such as during training sessions, multidisciplinary meetings, and/or discussions with the casemanager, in which minimal changes are required (other than refining goals) and being a matter of trial and evaluation. |
| Implementation | Experiences multidisciplinary meetings | Everything related to whether or not patients attend multidisciplinary meetings, the associated reasoning, challenges, and challenges and suggestions for improvement, such as the absence of therapists, difficulties in determining progress, attention to overlap between disciplines, and the need for deeper discussions. |
| Implementation | Information about PROMs | Everything related to the need for providing an explanation before using the measurement, emphasizing the necessity and importance based on research and patient needs, and outlining the procedure for uniform implementation. This should involve stakeholders (from the organization, former patients, researchers) to increase support for using the measurements. |
| Implementation | Added value: insights in individual progress | Everything related to the added value of measurements at the individual level for both patients and healthcare providers, as it provides an initial insight into the current state of rehabilitation and goals, as well as an assessment of whether adjustments or expansions are needed. |
| Implementation | Added value: insights in progress ILVR | Everything related to how measurements provide insight into the overall course of the rehabilitation, allowing individual rehabilitation to be compared with the average, identifying general stagnations in the programs, offering suggestions for adjustments, and contributing to the future viability and sustainability of ILVR. |
| Implementation | Lack of added value | Everything related to the lack of perceived necessity to modify the current measurements, with participants being satisfied with the current approach. |

*ILVR Inpatient low vision rehabilitation PROM patient-reported outcome measure*
